# Review of the tactical evaluation tools for youth players, assessing the tactics in team sports: football

**DOI:** 10.1186/s40064-015-1462-0

**Published:** 2015-11-02

**Authors:** Sixto González-Víllora, Jaime Serra-Olivares, Juan Carlos Pastor-Vicedo, Israel Teoldo da Costa

**Affiliations:** Teacher Training Faculty of Cuenca, University of Castilla-la Mancha (UCLM), Edificio Fray Luís de León, Campus Universitario s/n, C.P. 16071 Cuenca, Spain; Teacher Training Faculty, University Católica de Temuco (UCT), Temuco, Chile; Teacher Training Faculty of Toledo, University of Castilla-la Mancha (UCLM), Toledo, Spain; Centre of Research and Studies in Soccer, University Federal de Viçosa (UFV), Viçosa, Brazil

**Keywords:** Tactical awareness, Procedural tactical knowledge, Task constraints, Measurement, Game performance, Sport teacher training, Team sport, Soccer

## Abstract

For sports assessment to be comprehensive, it must address all variables of sports development, such as psychological, social-emotional, physical and physiological, technical and tactical. Tactical assessment has been a neglected variable until the 1980s or 1990s. In the last two decades (1995–2015), the evolution of tactical assessment has grown considerably, given its importance in game performance. The aim of this paper is to compile and analyze different tactical measuring tools in team sports, particularly in soccer, through a bibliographical review. Six tools have been selected on five different criteria: (1) Instruments which assess tactics, (2) The studies have an evolution approach related to the tactical principles, (3) With a valid and reliable method, (4) The existence of publications mentioning the tool in the method, v. Applicable in different sports contexts. All six tools are structured around seven headings: introduction, objective(s), tactical principles, materials, procedures, instructions/rules of the game and published studies. In conclusion, the teaching–learning processes more tactical oriented have useful tactical assessment instrument in the literature. The selection of one or another depends some context information, like age and level of expertise of the players.

## Background

One of the key objectives of sports assessment is the players’ ongoing training; therefore, emphasis should be placed on developing intelligent and creative players (Memmert 2010; Mitchell et al. 2006). An intelligent player is one who is capable of controlling the greatest possible number of technical-tactical variables in a short time and choosing the best possible option at all times during the game. While that creativity entails varying, rare and flexible decision-making in complex game situations (Memmert and Roth 2007).

Some of the variables which must be controlled in order to be an intelligent and creative player are, among others, space–time command, the different rhythms of the game, the scoreboard and timing of the match, the opponent’s strengths and weaknesses, one’s own limitations and the potential of the team during each play. These features are part of the player’s ability to adapt to the context of the game, known as tactical knowledge (González-Víllora et al. 2015).

Tactical knowledge is not inherent to players; it is developed and learned. Therefore, it must be assessed progressively throughout their training. Having excellent knowledge and specific experiences are the basis to making the right decisions quickly and being able to solve situations of different levels of uncertainty successfully.

The evaluation of observable tactical behaviour in athletes or players has been a study subject of great interest in recent years (Del Villar and García-González 2014; González-Víllora et al. 2015; Otero-Saborido and González-Jurado 2015). The analysis of decision making and the specific technical-tactical skills is essential to develop optimal and comprehensive training processes for athletes (González-Víllora et al. 2011, 2015). In invasion games, games played in a common area and simultaneous action on the ball (soccer, basketball, handball, hockey, etc.), it is necessary to measure the strategic aspects (Gutiérrez-Díaz et al. 2011). Therefore, we need to move away from the traditional teaching-evaluation approach in sports, focusing on sports technique. Currently, technique and tactics are considered two inseparable representations of a player’s actions. (García-López 2008). That is because it is important to adopt a more ecological approach when it comes to training and evaluating athletes.

Along this line, the use of observable assessment tools is common in sports research, since it allows us to analyze and describe the dynamics of the game (Gorospe et al. 2005). The aim of this research is to analyze and describe assessment tools capable of identifying and measuring tactical knowledge of soccer (real game) in a valid and reliable way.

## Method

The literature search was conducted in the period since 1995 until 2015. Therefore, the objective has been to have the evaluation tools of the past 20 years. A search was conducted in the following bibliographic databases: Dialnet Plus, EBSCOhost Online Research Databases, Emerald, MedLine, ISI Web of Knowledge, Science Direct y SportDiscus. The key words used were: “football/soccer evaluation tool/instrument/test”, “tactical evaluation/assessment”, “(procedural) tactical knowledge”, “tactical awareness”, “team sports evaluation”, and “game performance analysis”.

Out of the tools detected, the most relevant were selected according to the inclusion criteria established in the search. These criteria were:

1. That the assessment tools study and analyze those variables which influence practical tactical knowledge in soccer.
2. That the studies have an evaluation approach related to the tactical principles of the game (team sports, soccer), regardless of the principles it analyzes.
3. That the validity and reliability of the tools be established and published in scientific journals.
4. For the assessment tool to have been applied in the method of different published studies and subsequently, the quality and use of the tool be proven in the scientific field.
5. That the articles are made in different sporting contexts, whether recreational, educational or competitive, or a combination of them.

## Results

Table 1 describes the six tools for performance analysis of athletes with regard to the tactics of team sports, which meet all five criteria outlined in the method. All tools are based on the assessment of the tactical principles of the game. Therefore, it is a more ecological approach to game behaviour since the player’s performance is valued in terms of the contextual factors which affect his ability to adapt functionally to the specific situations in which he is assessed.

**Table 1:** Characteristics of the assessment tools of tactical knowledge in invasion sports

Name of the tool and acronym	Recom-mended age	Principles of performance being evaluated	Sports group being evaluated
Game performance assessment instrument (GPAI)	6–14 years of age	Score a goal (finishing): keep possession of the ball, attack the opponent’s goal, create space in attack and use the space in attack. Prevent your opponent from scoring: defend the space, defend the goal line and get the ball back Restart the game; throw the ball, corner kicks and free kicks	Invasion sports Basketball, handball, soccer, etc
Performance assessment in team sports (TSAP)	+12–13 years of age	Evaluates among other factors: received balls (RB), conquered balls (CB), offensive balls (OB), successful shots (SS), volume of play (PB) or lost balls (LB)	Soccer, basketball, handball, or volleyball
Procedural tactical knowledge test (KORA)	6–12 years of age	General principles: try to create numerical superiority, to avoid numerical equality and not to allow numerical inferiority	Invasion sports Soccer
Game performance evaluation tool (GPET)	6–14 years of age	Operational principles of play. Offensive: keep possession of the ball, advance towards the opponent’s field and score in the opponent’s goal. Defensive: regain possession of the ball. Prevent the opponent’s advance and protect your own goal and the opponent’s finishing	Invasion sports Basketball, handball or soccer
System of tactical assessment in soccer (FUT–SAT)	More than 11–12 years of age	Fundamental principles of play. Offensive: penetration, offensive coverage, width and length, depth mobility and offensive unity. Defensive: delay, defensive coverage, balance, concentration and defensive unity	Soccer and futsal
Game performance analysis	More than 16 years of age	Specific principles of each team (these principles are not defined since they are different for each team)	Basketball, handball, soccer, rugby or volleyball

The term tactical principle is used to refer to the contextual problems in a specific game situation. The set of maxims a player must keep in mind depending on the motor conditions he faces is seen as problems regarding game tactics or tactical principles. These principles establish the starting point, the basis; they represent the source of the action. They define the invariant properties on which the fundamental structure of the developments will be carried out (Bayer 1992).

Next, the tools shown in Table 1 are described following the order established by said table. Each one of them is divided into seven sub-sections: introduction, objective/s, tactical principles in which the following are developed: behaviour in play, materials, procedures, instructions and regulations of the type of game, assessment situation and studies in which the tool has been used.

In the literature, there are more invasion sports assessment tools. However, they have not been included to not meet any of the criteria outlined in the method. For example, an interesting tool can be the formative assessment of invasion games (Otero-Saborido and González-Jurado 2015), but this tool is very recent and therefore there is still no empirical studies (criteria 4).

## Game performance assessment instrument (GPAI)

### Introduction

This tool was developed in the USA by Oslin et al. (1998). It is a useful tool to evaluate youngsters from 6 to 14 years of age, both in the fields of education and research (Mitchell et al. 2006). The tool identifies the observable components of game performance, which can be applied to four categories of play: invasion sports, net and wall, aim and target, field and bat. Oslin et al. (1998) identified seven common components in the development of these four categories of play, such as base position, setting, decision making, execution skills, coverage, help/support and marking. Not all these components can be applied to a specific sport. In tennis 1 versus 1, for example, there is no player support. Thus, the coach/teacher must choose which of the seven components are the most significant in terms of what is to be taught and assessed. In 2008, Memmert and Harvey proposed some concerns and solutions for further development of GPAI.

### Objective/s

Assessment of the player’s decision making in invasion sports. Tactical behaviour in invasion sports can be measured in soccer, basketball, lacrosse and rugby, among others.

### Tactical principles evaluated

Some tactical principles which children should solve, depending on the learning stage, are selected. These principles are divided into three sections (Mitchell et al. 2006):

- Scoring: maintaining possession of the ball, attacking the goal, creating space in attack and using space in attack.
- Preventing scoring: defending space, defending goal and winning the ball.
- Restarting play: throw-in, corner kick and free kick (attacking and defending).

### Materials

A log sheet and a signature. For the field test, it is necessary to have cones, measuring tape, balls and goals. If you want to record the test, it is necessary to have a video camera on a tripod.

### Procedure

The GPAI is used for the assessment of actions and decisions of the players during a modified game practice, in which the rules, space, time and material are adapted, according to their skills. Usually, a game similar to that in the competition, or small-sided game, is played in order to keep the main characteristic and tactical essence of sport.

### Instructions and rules of the game

In soccer, a modified game has been selected. It is 4 versus 4, no goalkeepers, a 30 × 15 m field and it has small goals. In the game, a goal area is marked, surrounding the goal nets (2 × 2 m), where players cannot go in. It is played with a ball adapted to the players’ characteristics. Playing time is 2 4-min halves with a 3-min interval. Next, we will present a sample GPAI, in which three out of the seven possible components have been chosen for evaluation: decision making, execution and support. The evaluation criteria for the technical and tactical action in a pass are outlined for each of the three components.

The following tables details teaching sport concepts and skills and assessing outcomes. Table 2 describes components and criteria of Game Performance Assessment Instrument. Table 3 describes what look for support for invasion games. In addition, Table 4 is team sport assessment procedure for invasion games and Table 5 explains peer assessment rubric criteria for invasion games.

**Table 2:**
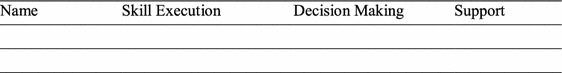
Components and criteria: GPAI for invasion games (Mitchell et al. 2006)

**Table 3:**
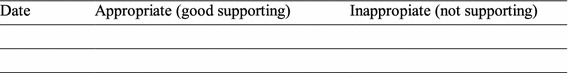
GPAI: support in invasion games (Mitchell et al. 2006)

**Table 4:**
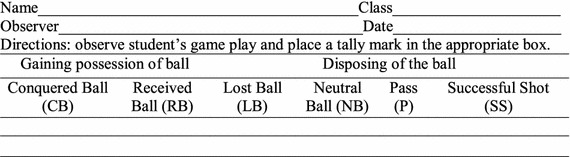
Team sport assessment procedure for invasion games (Mitchell et al. 2006)

**Table 5:**
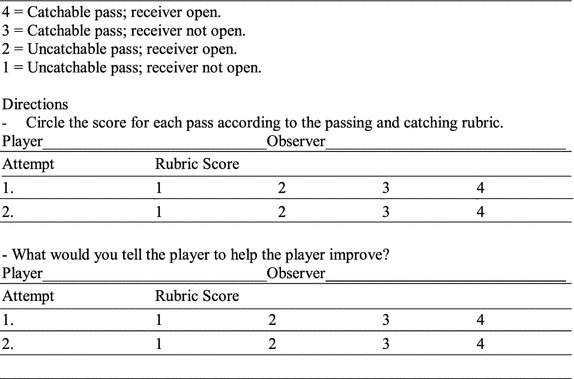
Invasion game: peer assessment rubric criteria (Mitchell et al. 2006)

GPAI for invasion games (Mitchell et al. 2006).

- Skill execution. Students pass the ball accurately, reaching the intended receiver.
- Decision making. Students make appropriate choices when passing (i.e., passing to unguarded teammates to set up a scoring opportunity).
- Support. Students attempt to move into position to receive a pass from teammates (i.e., forward toward the goal).

GPAI: Support in Invasion Games.

- What to look for

### Support

Students should attempt to move into position to receive a pass form a team-mate.

- Appropriate support

 Moving forward to space after pass is made.

 Positioning self in a passing lane.

 Moving quick and calling for the ball.

- Recording directions

 Read the three previous points about good support.

 Use a tally to mark each player’s attempt to support during the game.

### Studies in which it has been used

GPAI has been used for the evaluation of tactical learning related to different sports categories, such as net games (Griffin et al. 1995) or invasion games (Mitchell et al. 1995), or in studies with different samples and learning contexts (Griffin and Richard 2003; Harvey 2003).

## Performance assessment in team sports (TSAP)

### Introduction

The Performance assessment in team sports (TSAP) designed by Grehaigne et al. (1997) in France, is used for both the scientific and teaching fields. This tool takes into account the interactions between tactical and technical efficiency.

### Objective

The evaluation procedure is strictly game oriented and yields information reflecting both motor and tactical skills. The objective is to assess individual performance in team sports in contexts of pre-assessment and formative assessment. An authentic assessment procedure based on the observation of player’s actions during matches yielded two performance indices: the efficiency index and the volume of play (Grehaigne et al. 1997).

### Tactical principles evaluated

TSAP evaluates the elements that appear in Table 6.

**Table 6:** Relationships between observation items and types of information collected (Grehaigne et al. 1997)

Observation items	Information collected
Received balls (RB)	Involvement of the player in the team’s play
Conquered balls (CB)	Defensive capacities of the player
Offensive balls (OB)	Player’s capacity of making significant passes to his or her partners (offensive capacities)
Successful shots (SS)	Player’s offensive capacities
Volume of play (PB)	General involvement of the player in the game
Lost balls (LB)	A small number reflects in good adaptation to the game

### Materials

**Fig. 1:**
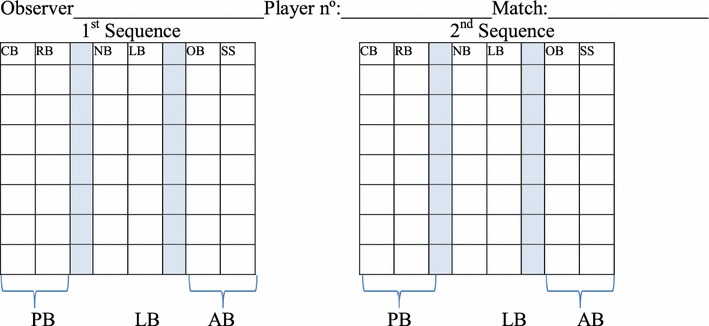
Observational grid for collecting raw data (Grehaigne et al. 1997). *CB* conquered ball, *RB* received ball, *NB* neutral ball, *LB* lost ball, *OB* offensive ball, *SS* successful shot, *PB* played balls

**Fig. 2:**
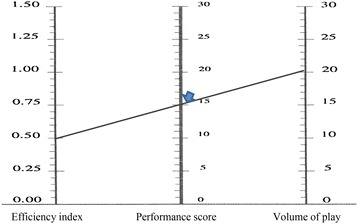
The monogram for assessing performance in team sports (Grehaigne et al. 1997)

There are two important materials; the first is an observational grid for collecting raw data (Fig. 1); the second is a monogram for assessing performance in team sports (Fig. 2).

The monogram for assessing performance in team sports is made of three different scales:

1. *The efficiency index scale* To build this scale, authors used samples totalling 302 senior high school students in different team sports (Basketball, European Handball, Soccer), and authors found that the efficiency index rarely exceeded 1.5. They have chosen to keep the same scale for different sports (0–1.5, with 30 equal intervals). If one player obtains an efficiency index value higher than 1.5, the 1.5 value is used.
2. *The volume of play scale* Authors have retained a scale ranging for 0–30, with 30 equal intervals.
3. *The performance score scale* This scale has been established on the basis of the following formula:

Performance score = (efficiency index × 10) + (volume of play/2).

The scale ranges from 0 to 30, with 30 equal intervals.

### Procedure

A first step is to observe a player during a match and registering various occurrences in order to establish two complementary performance indices: the efficiency index and the volume of play. The observational sheet is constructed so that each row should contain two marks: one to indicate how the player gained possession of the ball, and one to indicate how the player disposed of the ball.

The player may gain possession of the ball in one of two ways:

1. Conquering the ball (CB). A player is considered having conquered the ball if he or she intercepted it. Stole it form an opponent, or recaptured it after an unsuccessful shot on goal or after a near-loss to the other team.
2. Receiving the ball (RB). The player receives the ball form a partner and does not immediately lose control of it.

The player may dispose of it in one of four ways:

1. Playing a neutral ball (NB). A routine pass to a partner or any pass which does not truly put the other team in jeopardy is considered a neutral ball.
2. Losing the ball (LB). A player is considered having lost the ball when he or she loses it to the other team without having scored a goal.
3. Playing an offensive ball (OB). An offensive ball is a pass to a partner which puts pressure on the other team and, most often, leads to a shot at goal.
4. Executing a successful shot (SS). A shot is considered successful when it scores or possession of the ball is retained by one’s team.

After the observer computes the total number for CB, RB, LB, OB and SS. These produce two additional pieces of information:

1. The number of attack balls (AB). An attack ball results from an offensive ball (OB) or from successful shot on goal (SS). AB = OB + SS.
2. The volume of play (PB). The volume of play represents the number of times the players has gained possession of the ball (PB, for played balls). PB = CB + RB.
3. The performance score is computed on the basis of two indices:

Efficiency index = (CB + AB)/(10 + LB) or (CB + OB + SS)/(10 + LB).

### Instructions and play/game rules

The assessment procedure was intended for older students (over 12 or 13 years old). Its integration to the teaching–learning process (with its limits of time and space, and its requirements of learning opportunities [ball exchanges]) and the desire to come up with one single procedure applicable to different sports made it necessary to look for appropriate modifications of each game (Grehaigne et al. 1997). It is therefore suggested that the matches be played under the following specific conditions.

- Basketball: Four players against four players on a regular court; two 7-min matches are played.
- European Handball: Five players (4 + 1) against five players (4 + 1) on a regular court; two 7-min matches are played.
- Soccer: Five players (4 + 1) against five players (4 + 1) on a 50 m × 30 m surface with 6 m × 2 m goals; regular soccer rules are applied with a few adjustments (e.g., “throw in” is done by foot, corners are done by hand, there is no “off side,” for dead balls or “free kicks,” opponents are placed at 6 m); two 7-min matches are played.

### Studies in which it has been used

TSAP has been used in different teaching–learning contexts with subjects of different ages and levels. A performance evaluation has been allowed, according to the tactical essence of sports such as soccer and other team sports (Gréhaigne et al. 2005; Richard et al. 2000).

## Procedural tactical knowledge test (KORA)

### Introduction

The Procedural Tactical Knowledge Test (KORA) was proposed by German researchers (Kröger and Roth 2002), and validated by Memmert (2002). KORA allows for the evaluation of tactical performance in all collective sports games, evaluating two parameters inherent to tactical abilities: positioning and movement (P.O.) and recognizing spaces (R. S.). The first parameter refers to the player`s ability to get the optimum position at the right time. The second one corresponds to the player’s ability to identify and develop opportunities to score a goal (Kröger and Roth 2002). Memmert (2010) proposed a test which analyzes a game with the ball, the actions of the teammates and the actions of defending players. The biggest drawback about this test is that the patterns of play are not standardized to measure tactical behaviour. Next, we preset a sample KORA: 3 versus 3.

### Objective/s

To determine the level of intelligence and tactical creativity in invasion games. Basic tactical motor behaviour is measured regarding the search for the ideal space–time situation at all times during the game and knowing when the best opportunities arise to score a goal.

### Tactical principles evaluated

General principles: to try to create numerical superiority, to avoid numerical equality and not to allow numerical inferiority.

### Materials

A video camera on a tripod, balls, measuring tape and cones to delimit the space in every playing field, log sheets, clips to mark the actions (plays) evaluated, six coloured chest guards with big numbers to identify the subjects in the video and two timers, one for the assessor and another one for the cameraman. It is necessary to have two people trained to carry out the test, one to operate the camera and another to carry out the protocol for the assessment of the game.

### Procedure

The first task is the installation of the playing field. It is a square (9 × 9 m) delimited by cones at the four corners. The assessor will give the players the instructions for the test and then he will ask questions to make sure everyone understands. Then, the players will play the game to get familiar with it. If the players break the rules during the game, the game and the recording will be stopped. If that happens, the process would start again, explaining the rules, until they have been understood. The person in charge of the video camera should be located in an area that enables all four corners of the field to be recorded, without having to move the camera. The distance is 3 m from the corner of the playing field, which would help the camera to be in an elevated position to make recording easier. The person recording will also keep track of time.

### Instructions and rules of the game

**Fig. 3:**
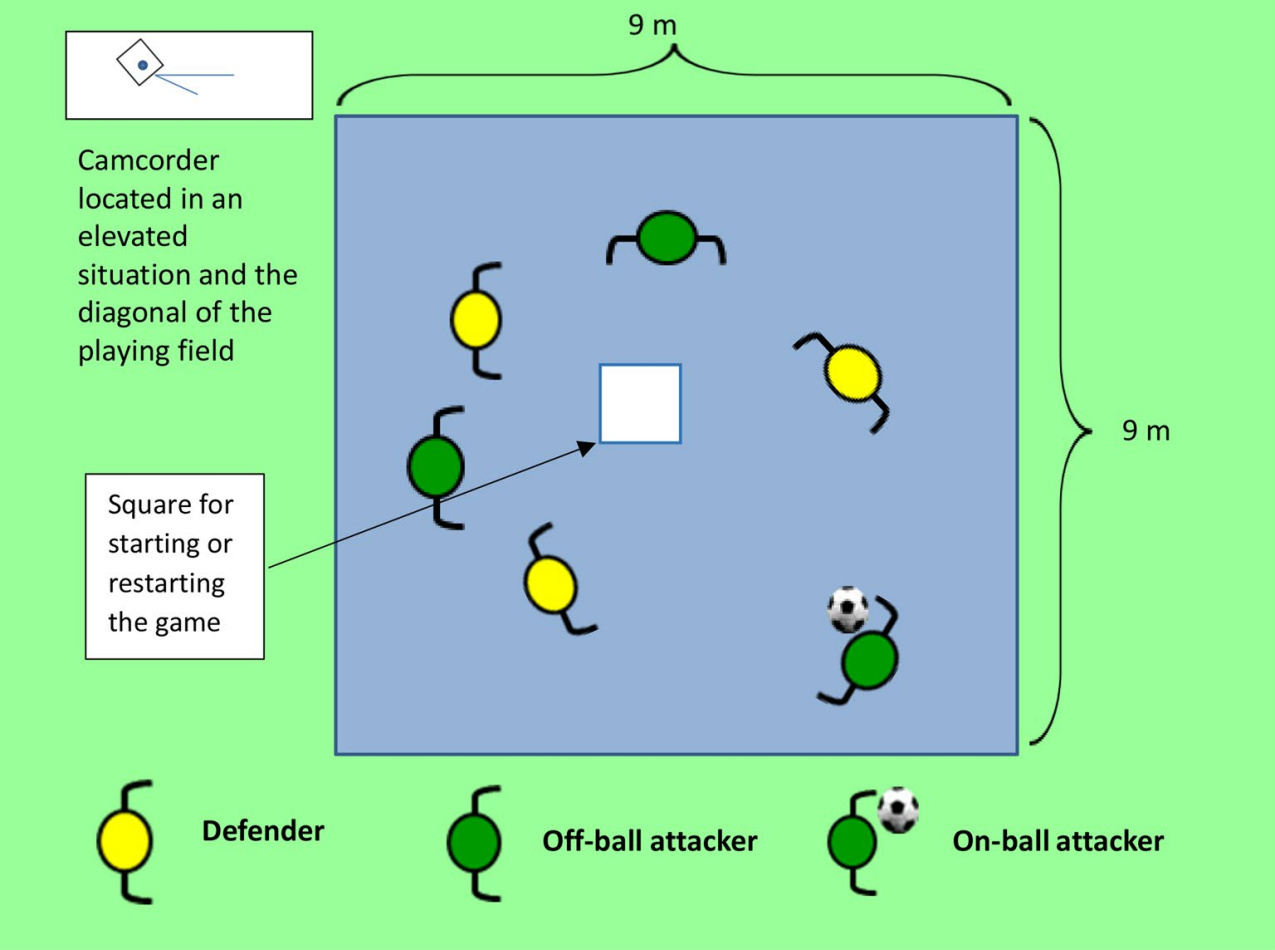
Graph explaining the KORA play situation 3 versus 3

A protocol is followed to ensure the correct use of the tool (Fig. 3).

1. The game lasts 3 min.
2. The aim is to pass without the defender intercepting the ball.
3. When the defender intercepts a pass, the game starts over from the centre of the play area. This time, the attacking team will be the one which has stolen the ball.
4. The defenders cannot grab the opponent, steal the ball if he is holding it with both hands or take it from his feet if he is stepping on it.
5. The attackers can move freely around the area, with or without the ball.
6. There will be someone to retrieve the balls which leave the area. If there is no one else, the assessor will do it. For the first pass, the defender must keep a minimum distance of 2 m.
7. Use the soccer rules regarding: drive, pass and dribble.

Studies in which it has been used KORA has been used in different teaching–learning contexts and in research, especially in soccer. It was implemented with samples in Germany (Memmert 2002, 2010) and Brazil (Aburachid et al. 2013; Giacomini et al. 2011).

## Game performance evaluation tool (GPET)

### Introduction

The Game Performance Evaluation Tool (GPET) was designed by García-López et al. (2013) in Spain. This tool provides the opportunity to analyze each decision made during the game from a tactical point of view of the problem of mobility which the player faces at all times during the game. This approach allows for a more ecological assessment of decision making in sports than the one adopted in previous decision-making assessment tools in games, such as GPAI (Oslin et al. 1998) or TSAP (Grehaigne et al. 1997). It should be noted that both these tools analyze decision making and skill execution, but they do not take into account specific tactical problems in game situations. GPET evaluates game performance at two different levels. The first level evaluates how the players’ actions adapt to the tactical principles (Bayer 1992): keeping possession of the ball, advancing towards the opponent’s goal and scoring a goal. At the second level, GPET separates the cognitive components from the decision making and motor skills.

### Objective/s

Evaluate decision making and skills execution in invasion sports.

### Tactical principles evaluated

Operating tactical principles: offensive (keeping possession of the ball, advancing towards the opponent’s goal and finishing; (see Table 7); defensive (regaining possession of the ball, preventing your opponent’s advance and protecting your own goal and the opponent’s finishing).

**Table 7:** GPET: game variables measured

Game roles	Individual technical-tactical element	
	Evaluated game principles	Decision making and success in the execution are measured
On-ball attacker	1A: Keeping 2A: Progressing 3A: Achieving the objective	Control (only execution is measured)
		Pass
		Carrying the ball/dribbling
		Shooting/finishing
Off-ball attacker	1A: Keeping 2A: Progressing	Losing one’s defender (get away)
		Fixing
Defender to on-ball attacker	–	Marking, pursuit or basic position
		Defensive blocking
		Tackle
		Clearing the ball
		Help
Defender to off-ball attacker	–	Marking, pursuit or basic position
		Interception
		Clearing the ball
		Helping the JDAcB

### Materials

A video camera on a tripod. Cones (40 units). Two small goals (95 × 70 cm.) and two large goals (140 × 105 cm), both detachable. Three footballs A-7 (63.5–66 cm). Fourteen chest guards with big numbers on the front and the back (from one to fourteen), half of them one colour and the other half, a different colour. Two whistles and two timers, one for the referee and the other one for the person in charge of the video camera. A 50 m measuring tape. In addition, the evaluation criteria and a log sheet are needed. They are shown in Tables 8 and 9.

**Table 8:** GPET. Assessment criteria for the off-ball attacker: losing one’s defender

Off-ball attacker	Decision making *Appropriate decisions* (1) The player tries to Occupy/stay in a free area, at an appropriate passing distance and in passing line Make a feint, creating a passing line *Inappropriate decisions* (0) The player Occupies a position close to an opponent Occupies the penetration space of a partner with ball Is static, marking, and does not allow a pass Commits an offense: offensive foul or stepping into a prohibited area (goal area) Is situated at an inadequate distance for the passer’s possibilities
	Execution *Successful executions* (1) Leave his marker behind Adopts a free position on a possible free pass lane *Unsuccessful executions* (0) Does not get away from his marker Remains static and does not allow for a pass from teammate when there is an opportunity

*Note* It is understood not to be necessary to be getting away from markers continuously, but it is necessary when a partner needs it or when the player is marked

**Table 9:** GPET. Record sheet: two roles of attacking play

Action/time	Principle			On-ball attacker					Off-ball attacker				
Nº	Min-sec.	Situation	Application		C Su	Decision making				Su	Decision making		Su
						P	C&D	S			Ofen. V	LOD	
1.													
2.													
Situation: Ideal tactical principle depending on situation Application: Tactical principle applied by the player Su: Execution. Success or lack of success in the skill execution							C Su: control success P: pass C&D: Carrying the ball/dribbling S: shoot LOD: Losing one’s defender (get away) V. Ofen.: offensive variables						

This tool permits carrying out simpler evaluation worksheets (Table 10), allowing the use of peer assessment or assessment between pairs of players.

**Table 10:** Off-ball attacker technical-tactical observation checklist: getting away

Watch a player who is playing and evaluate the following items					
Observer	1	2	3	4	5
He keeps at a proper distance from the attacking player with the ball					
He is very close to other players from the same team that do not have the ball					
When moving, player goes to a space where there is direct passing line with the on-ball player					
He is usually well marked or unmarked					
When he is in possession and passes, he moves quickly to a free space					

### Procedure

First action to be taken, is marking the field with bright-coloured cones. Fields will be previously marked with the proper measurements for each training category or academic year (Table 11).

**Table 11:** Game features modified by age and number of players per team

Age (years)	Nº of players	Time nº × min	Field playing area m × m	Goal area m × m	Goals measurements cm × cm
Under-8	2 × 2	2 × 4′	1/8 of field A-7 (20 × 10)	3 × 4	95 × 70
	3 × 3		1/4 of field A-7 (32 × 22)	5 × 9	140 × 105
Under-10	3 × 3	2 × 4′	1/4 of field A-7 (32 × 22)	5 × 9	140 × 105
	4 × 4		1/2 of field A-7 (44 × 32)	7 × 14	140 × 105
Under-12	3 × 3	2 × 4′	1/4 of field A-7 (32 × 22)	5 × 9	140 × 105
	4 × 4		1/2 of field A-7 (44 × 32)	7 × 14	140 × 105
	5 × 5		3/4 of field A-7 (52 × 40)	11 × 24	140 × 105
Under-14	7 × 7	2 × 4′	Soccer field A-7	Goal area A-7	Soccer goal A-7

Two teams of players, designated by the teacher/trainer, will be organized based on the nature and level of the players. They will be organized in such a way that all teams are as balanced as possible. Players who are going to be recorded should practice the same game as in the assessment a week before, in order to become familiar with the presence of the camera. For recording, the position of the camera will be behind the baseline, with enough space to record with high quality and record the whole field without moving the “zoom” (5 m long y 8 m wide maximum). The recording will not be interrupted other than at halftime, where there will be a change of fields.

### GPET instructions and play/game rules

The game will last two parts of 4 min, with a 3-min break in between. There will not be stoppage time for turnovers, and the stopwatch will not be stopped when there is a violation of the rules. Each part will finish after the 4 min. It is mandatory for players of the team without possession of the ball to make individual defense, always marking the same opposing player, unable to use any other type of defensive tactics. If the defender invades the defending goal area and the attacker whom he is defending has not passed yet, a foul will have been committed. This foul will restart with a throw-in at the nearest point to the foul. The attackers will be able to invade goal areas defended by the opposing team without fouling. You cannot shoot on opposite goal from your own field. All fouls whistled are indirect. If in doubt at any part of the game, the official rules of the Spanish soccer Federation for A-7 will be used.

### Studies where it has been used

This tool has been used in different contexts of teaching and learning of sport, such as: (1) academic (Sánchez-Mora et al. 2011); (2) recreational-competitive, where the effectiveness of small-sided games of representation and exaggeration with the operational principles have been compared (e.g., Serra-Olivares et al. 2015a, b); (3) competitive, where different training categories have been assessed (e.g., U10 football players: González-Víllora et al. 2011), or the evolution of tactical knowledge in players with high level of expertise in soccer has been assessed: since U8 to U14 players (González-Víllora et al. 2015); (4) the combination of academic and competitive area, where there have been comparisons between expert and novice players of the same age (Gutiérrez-Díaz et al. 2011).

This tool has been adapted to net sports, as an example studies in squash: the validation of the tool: Squash Performance Evaluation Tool (Catalán-Eslava and González-Víllora 2015) and analysis of execution and visual search behavior on return action (Catalán-Eslava et al. 2014).

## System of tactical assessment in soccer (FUT–SAT)

### Introduction

The System of Tactical Assessment in Soccer (FUT–SAT) was developed in a partnership between Portugal and Brazil (Teoldo et al. 2011). The purpose of the system is to provide a method for coaches, teachers and researchers to access specifically and objectively the information that reflect tactical behaviors performed by players in actual match context. Its conceptual structure is based on ten core tactical principles of Soccer. The rationale for the selection of these principles is supported by their representation of the core aspects of the process of teaching and training of tactical skills. Besides this, this set of principles provides objective measures of players’ motion with respect to their management of the playing space. FUT–SAT provides information about the tactical behavior, tactical performance and decision making of each player in the game (Teoldo et al. 2009). The authors suggest the application of the test with players over 11–12 years old, since children need to have their cognitive processes developed to allow them to think abstractly in order to play according to the core tactical principles.

**Fig. 4:**
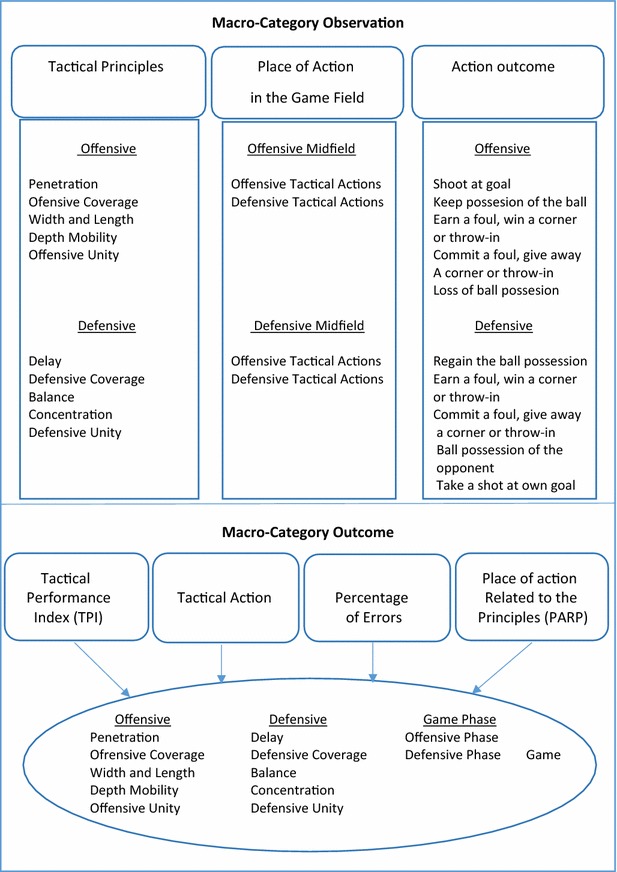
Structural organization of FUT–SAT’s variables

FUT–SAT is structured according to the class of data the system deals with and is comprised by two macro-categories, seven categories and 76 variables (see Fig. 4). The Macro-Category Observation comprises three categories and 24 variables. The Macro-Category named Tactical Principles includes ten variables. The category Place of Action in the Game Field contains four variables while the category Action Outcomes comprises ten. The other Macro-Category, Outcome, comprises four categories and 52 variables. In this Macro-Category, all four categories—Tactical Performance Index (TPI), Tactical Actions, Error Percentage and Place of Action Related to the Principles (PARP)—comprise the same thirteen variables. The Macro-Category Outcome is so called once its variables depend on the information that derive from the variables of the Macro-Category Observation.

### Objective/s

Assessment of the tactical behavior of Soccer and Futsal players.

### Tactical principles assessed

Offensive phase: penetration, offensive coverage, mobility, space and offensive unity. Defensive phase: delay, defensive coverage, balance, concentration and defensive unity (see Fig. 4).

### Materials

A video camera placed on a tripod, seven soccer balls (size n. four) for children up to 10 years of age and n. five for children aged 11 or more, straps to indicate the dimension of the goal and playing areas, a timer, tape measure, numbered and different coloured vests, small goals (or poles and two straps to emulate goal posts).

### Procedure

**Fig. 5:**
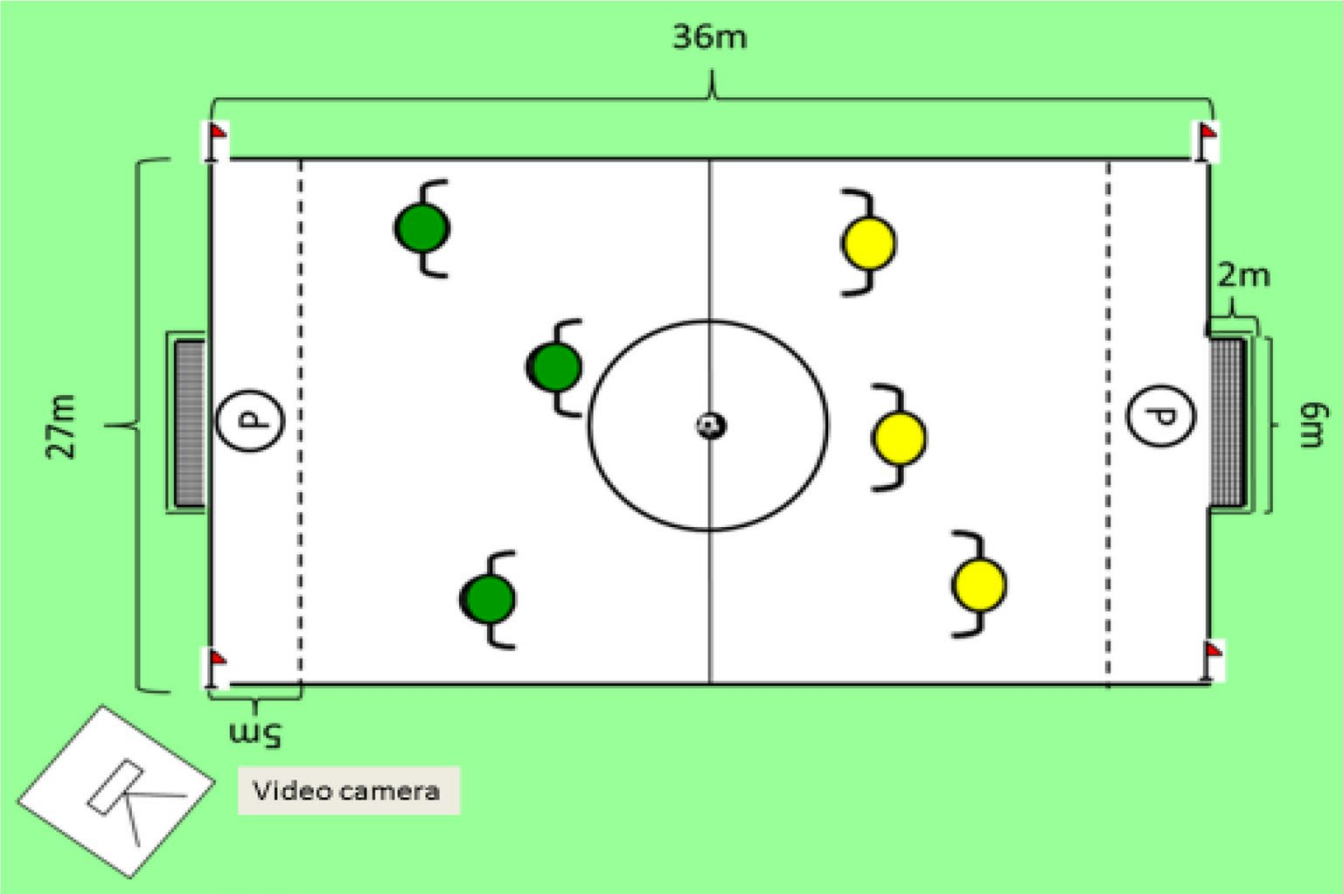
3 versus 3 game situation in FUT–SAT

**Fig. 6:**
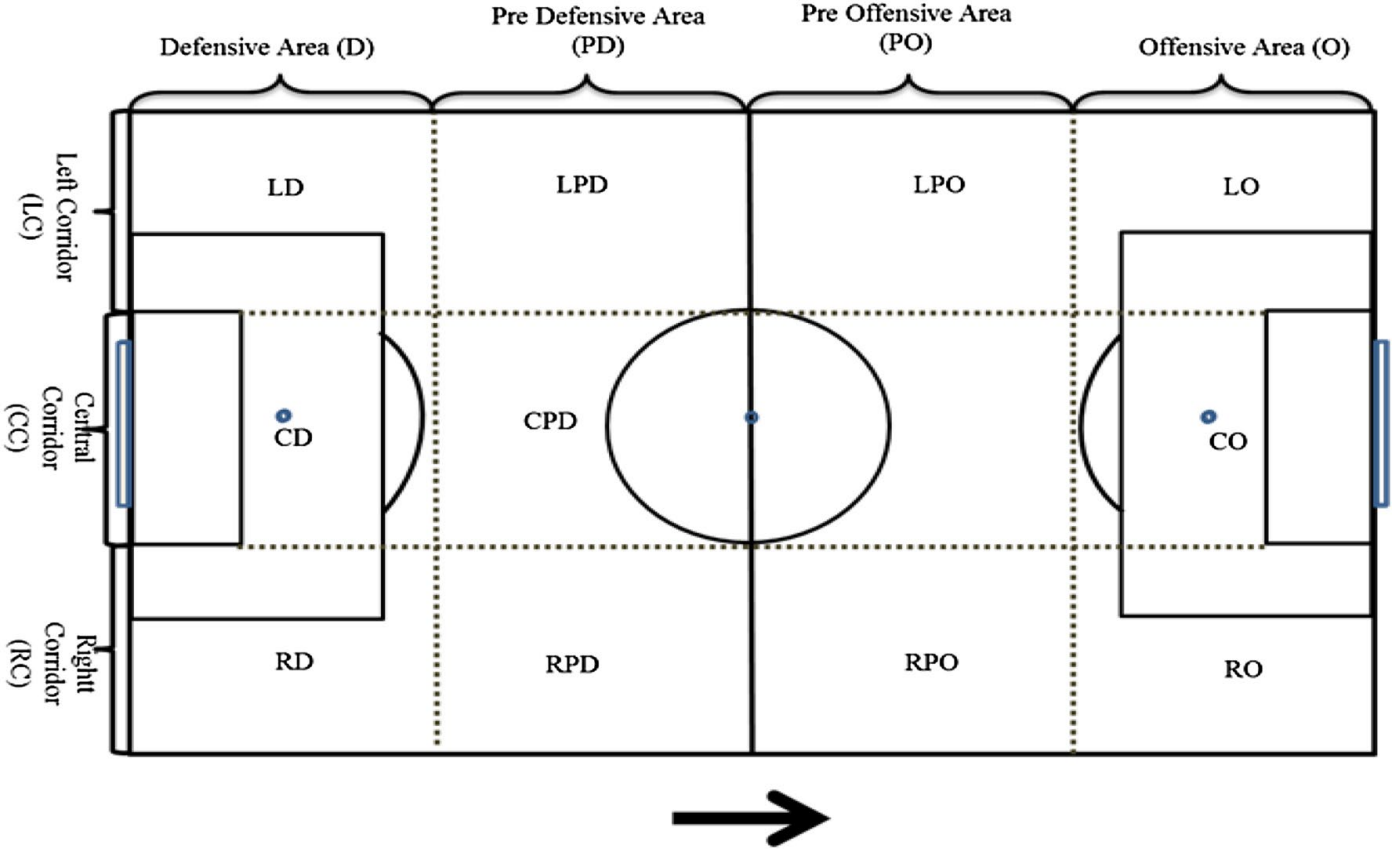
Representation of the physical structure of FUT–SAT’s field test

The field test of FUT–SAT may include one goalkeeper and three outfield players (GK + 3 vs. 3 + GK) up to one goalkeeper and ten outfield players for both teams (GK + 10 vs. 10 + GK). The dimensions of the field in this test were calculated based on the number of players, and the dimensions of a Soccer field specified by the International Football Association Board and on the ratio calculation of the utilization of playing space by the outfield players. The standard field test is named “GK + 3 versus 3 + GK” Test, and is performed during 4 min in a field of 36 m long by 27 m wide (Fig. 5). Experts must provide exactly the same information about the test to all participants, in order not to influence results because of this issue. Two experts are necessary for conducting the test. Their tasks are: the person applying the test should delimit the area of play and conduct the test. Before the start of the test, the second person, who is responsible for handling the videocamera, should apply the zoom in order to focus on the faces and numbers of the players to have them identified. He/she should write down the date, game and test number, with studying objective. Figure 6 includes the representation of the physical structure of FUT-SAT’s game analysis.

### FUT–SAT’s instructions and playing rules

The following information to assess players is provided: “You are going to play a small-sided game, named “GK + 3 versus 3 + GK”, in which the execution of the tactical principles will be assessed. This test is comprised by two parts of 4 min each. The goalkeeper is only allowed to play inside the penalty area (5 m), and cannot leave this delimited space. Official soccer rules will apply. After each goal, the game restarts with the goalkeeper and not from the midfield”. (1) A ball boy must be placed at each end of the field, to facilitate the replacement of balls as fast as possible. (2) Once the first 4 min are over, the teams will change sides and resume play (defense-attack). (3) Before the start of the test, the six players and two goalkeepers will be repositioned so as to be identified in the video analysis.

### Studies that have utilized FUT–SAT

Since its design and validation (Teoldo et al. 2009, 2011), the tool has been used in different contexts in soccer studies (Castelão et al. 2014; Gonzaga et al. 2014; Moraes et al. 2014; Silva et al. 2014).

## Game performance analysis or match analysis

The game analysis for observing the behaviour of teams and players started a long time ago (Reep and Benjamin 1968) and has been changing during the time in order to provide quick and useful for coaches and players (Garganta 2001). Since the beginning of decade 2000 and in the early days, researchers have highlighted dynamics aspects of the game in order to have richer and applicable information about the players behaviour’s on the pitch. This sort of information have been acceptable for enhance performance in youth and professional teams. In these cases the assessment of performance, especially the tactical assessment, is performed by the most advanced technology (SPORT CODE or AMISCO), following the parameters and criteria of game analysis.

The game analysis can comprehends three phases: (1) events observation; (2) data annotation; and (3) data interpretation (Hughes and Franks 2004). The resulting data allows identification of critical factors and elements that influence the performance of teams and players (Garganta 2001; Lago 2009). In general, game analysis permits the recording of recent information, to implement improving tasks now and progress in the future (Hughes and Franks 2004).

Some examples on the game analysis of teams that have recently highlighted by its performance can be: Barcelona team’s performance and his opponents in the final games of the Champions League and the FIFA Club World Cup 2010 (Cambre-Añon et al. 2014), analysis of the offending patterns of Spain national soccer team in FIFA World Cup 2010 in relation to the status of the match (Moraes et al. 2014), measuring collective behaviour in Football teams: inspecting the impact of each half of the match on ball possession (Clemente et al. 2013), or analyzed the network characteristics of successful and unsuccessful national teams that participated in FIFA World Cup 2014 (Clemente et al. 2015).

## Conclusions

Tactical performance assessment should be attached firmly to the teaching–learning process. That is, if the new processes of youth training are based on the strategy, cognitive-motor player involvement, tactical game principles problem solving; the assessment should follow the same line, assessing the degree of adaptation of the players on tactical problems of all phases of the game: offense, defense, attack, counter-attack or defensive withdrawal.

For a proper and effective tactics assessment, quality measurement instruments are required. In sport initiation (from approximately 6–8 to 12–14 years of age), there are several assessment tools that assess the performance of the players in relation to the tactical principles, such as GPAI, GPET, KORA, o TSAP at the end of this stage. Teachers/coaches are advised to select at least one of these tools to assess the progress of children, taking into account the inner practice context: class or training contents, tactical and technical level of players and the knowledge of the observer-assessor evaluation tools, as some tools are more complex than others in their procedures. The more complex it is, the more time will be necessary to perform the results analysis. Therefore, GPAI is the easiest to use, thus more practical for academic education. KORA and TSAP are at an intermediate level. While GPET is the tool with a slightly more complex procedure, but in turn provides more information than the rest, as the technical and tactical elements assessment are linked to the operational principles: progress and implementation of the player at all times (Bayer 1992), which is a more ecological application on the tactical assessment. All four tools are suitable for the educational and scientific field.

From 12 years of age, players manage to think abstractly and develop more refined tactical group behaviour during learning practice of situations closest to federated sport (González-Víllora et al. 2015; Gutiérrez-Díaz et al. 2011). Therefore, the difficulty of learning and of the motor behaviour to be developed increases and so does the complexity to assess these variables. The criteria to assess tactical knowledge regarding the demands of the game must be made in accordance with the most advanced and specific principles. With this in mind, the assessment based on the fundamental tactical principles seems to be the best alternative, with the FUT–SAT as the most recommended tool.

To be used, all tools presented in this work require a minimum of prior training for evaluators-observers. This training must be conducted by an expert in the procedures and implementation of each tool. Later, the expert must measure the intra and inter reliability of the observer. Once both variables are positive, the new assessor is able to measure the assessment tool. All tools presented in this work are useful, easy to use and relevant for assessment of gaming performance in games and team sports.

As prospective, it would be very interesting to carry out studies with several of the tools included in the method, in order to cross the results, obtaining more valuable results and discussions.

## References

[CR1] Aburachid LMC, da Silva SR, Greco PJ (2013). Tactical knowledge level players and soccer coach´s subjective evaluation. Revista Brasileira de Futsal e Futebol.

[CR2] Bayer C (1992). La enseñanza de los juegos deportivos colectivos [The teaching of collective sports games].

[CR3] Cambre-Añon I, Lizana CJR, Calazans E, Machado JC, Teoldo I, Scaglia AJ (2014). Performance of Barcelona`s team and their opponents in the finals matches of the Champions League and the FIFA Club World Cup 2010. Revista Andaluza de Medicina del Deporte.

[CR4] Castelão D, Garganta J, Santos R, Teoldo I (2014). Comparison of tactical behaviour and performance of youth soccer players in 3v3 and 5v5 small-sided games. Int J Per Anal Sport.

[CR5] Catalán-Eslava M, González-Víllora S (2015). Validation of a wall-net sports measurement instrument: squash performance evaluation tool (SPET). Retos.

[CR6] Catalán-Eslava M, González-Víllora S, Abellán-Hernández J, Contreras-Jordán OR (2014). Analysis of execution and visual search behavior on return action in Squash. Cultura, Ciencia y Deporte.

[CR7] Clemente FM, Couceiro MS, Martins FML, Mendes R, Figueiredo AJ (2013). Measuring collective behaviour in Football teams: inspecting the impact of each half of the match on ball possession. Int J Perform Anal Sport.

[CR8] Clemente FM, Martins FML, Kalamaras D, Wong DP, Mendes RS (2015). General network analysis of national soccer teams in FIFA World Cup 2014. Int J Perform Anal Sport.

[CR9] Del Villar F, García-González L (2014). El entrenamiento táctico y decisional en el deporte [The decisional and tactical training in sport].

[CR10] García-López LM (2008) Research and teaching of techniques and tactics in invasion games. Implementation in Soccer. Cultura, Ciencia y Deporte 3(9):161–168

[CR11] García-López LM, González-Víllora S, Gutiérrez-Díaz D, Serra-Olivares J (2013). Development and validation of the game performance evaluation tool (GPET) in soccer. Revista Euroamericana de Ciencias del Deporte.

[CR12] Garganta J (2001). The analysis of performance in sports games. Revisão Acerca da Análise do Jogo.

[CR13] Giacomini DS, Soares VO, Santos HF, Matias CJ, Greco PJ (2011). Declarative and procedural tactical knowledge in soccer players of different ages. Motricidade.

[CR14] Gonzaga A, Albuquerque MR, Malloy-Diniz LF, Greco PJ, Teoldo I (2014). Affective decision-making and tactical behaviour of Under-15 soccer players. PLoS One.

[CR15] González-Víllora S, García-López LM, Pastor-Vicedo JC, Contreras-Jordán OR (2011). Tactical knowledge and decision making in young football players (10 years old). Rev Psicol Depor.

[CR16] González-Víllora S, García-López LM, Contreras-Jordán OR (2015). Decision making and skill development in youth football players. Int J Med Sci Phys Act Sport.

[CR17] Gorospe G, Hernández A, Anguera MT, Martínez R (2005). Development and optimization of an observational tool for singles tennis. Psicothema.

[CR18] Grehaigne JF, Godbout P, Bouthier D (1997). Performance assessment in team sports. J Teach Phys Educ.

[CR19] Gréhaigne JF, Richard JF, Griffin L (2005). Teaching and learning team sports and games.

[CR20] Griffin L, Richard JF (2003). Using authentic assessment to improve students’ net/wall game play. Teach Elem Phys Educ.

[CR21] Griffin L, Oslin J, Mitchell S (1995). An analysis of two instructional approaches to teaching net games. Res Q Exerc Sport.

[CR22] Gutiérrez-Díaz D, González-Víllora S, García-López LM, Mitchell S (2011). Differences in decision-making between experienced and inexperienced invasion games players. Percept Motor Skill.

[CR23] Harvey S (2003) Teaching games for understanding: a study of U19 college soccer players improvement in game performance using the game performance assessment instrument. In 2nd international conference: teaching sport and physical education for understanding. University of Melbourne, Australia

[CR24] Hughes M, Franks I (2004). Notational analysis of sport: systems for better coaching and performance in sport.

[CR25] Kröger C, Roth K (2002). Escola da bola: Um ABC para iniciantes nos jogos esportivos [School ball: An ABC Sports games for beginners].

[CR26] Lago C (2009). The influence of match location, quality of opposition, and match status on possession strategies in professional association football. J Sports Sci.

[CR27] Memmert D (2002) Diagnostik taktischer leistungskomponenten: Spieltestsituationen und konzeptorientierte expertenratings [Tactical performance components valuation: test situations and concept-oriented expert ratings]. Ph.D. Dissertation. Heidelberg University, Heidelberg

[CR28] Memmert D (2010). Testing of tactical performance in youth elite soccer. J Sports Sci Med.

[CR29] Memmert D, Harvey S (2008). The game performance assessment instrument (GPAI): some concerns and solutions for further development. J Teach Phys Educ.

[CR30] Memmert D, Roth K (2007). The effects of non-specific and specific concepts on tactical creativity in team ball sports. J Sports Sci.

[CR31] Mitchell S, Griffin L, Oslin J (1995). An analysis of two instructional approaches to teaching invasion games. Res Q Exerc Sport.

[CR32] Mitchell SA, Oslin JL, Griffin L (2006) Teaching sport concepts and skills. A tactical approach (2 ed). Human Kinetics, Champaign, IL

[CR33] Moraes EL, Cardoso F, Teoldo I (2014). Análise dos padrões ofensivos da seleção espanhola de Futebol na Copa do Mundo FIFA 2010 em relação ao status da partida [Analysis of the offending patterns of Spain national football team in FIFA World Cup 2010 in relation to the match status]. Revista Brasileira de Educação Física e Esporte.

[CR34] Oslin JL, Mitchell SA, Griffin LL (1998). The game performance assessment instrument (GPAI): development and preliminary validation. J Teach Phys Educ.

[CR35] Otero-Saborido FM, González-Jurado JA (2015). Design and validation of a tool for the formative assessment of invasion games. J Phys Educ Sport.

[CR36] Reep C, Benjamin B (1968) Skill and chance in association football. J Royal Stat Soc. Series A (General) 131(4):581–585

[CR37] Richard JF, Godbout P, Gréhaigne JF (2000). Students’ precision and reliability of team sport performance. Res Q Exerc Sport.

[CR38] Sánchez-Mora D, García-López LM, Del Valle MS, Solera I (2011). Spanish primary school students’ knowledge of invasion games. Phys Educ Sport Pedag.

[CR39] Serra-Olivares J, González-Víllora S, García-López LM (2015). Effects of the modification of task constraints in 3 vs. 3 small-sided soccer games. S AFR J Res Sport, Phys Educ Recreat.

[CR40] Serra-Olivares J, González-Víllora S, García-López LM, Araújo D (2015). Game-centred approaches’ pedagogical principles: exploring task constraints in youth soccer. J Hum Kinet.

[CR41] Silva B, Garganta J, Santos R, Teoldo I (2014). Comparing tactical behaviour of soccer players in 3 vs. 3 and 6 vs. 6 Small-Sided Games. J Hum Kinet.

[CR42] Teoldo I, Garganta J, Greco PJ, Mesquita I (2009). Tactical principles of soccer game: concepts and application. Motriz.

[CR43] Teoldo I, Garganta J, Greco PJ, Mesquita I, Maia J (2011). System of tactical assessment in soccer (FUT-SAT): development and preliminary validation. Motricidade.

